# Easing batch image processing from OMERO: a new toolbox for ImageJ

**DOI:** 10.12688/f1000research.110385.2

**Published:** 2022-09-12

**Authors:** Pierre Pouchin, Rayan Zoghlami, Rémi Valarcher, Maxence Delannoy, Manon Carvalho, Clémence Belle, Marc Mongy, Sophie Desset, Frédéric Brau

**Affiliations:** 1 GReD, CNRS, INSERM, Université Clermont Auvergne, Clermont-Ferrand, France; 2 Université Côte d’Azur, CNRS, IPMC, Valbonne, France; 3 Polytech Nice Sophia, Campus SophiaTech, Sophia Antipolis, France; 4 Univ. Lille, CNRS, Inserm, CHU Lille, Institut Pasteur de Lille, U1019 - UMS 9017 - CIIL - Center for Infection and Immunity of Lille, Lille, 59000, France

**Keywords:** OMERO, Fiji, ImageJ, Java, Image Analysis, Image processing, Automation, Microscopy

## Abstract

The Open Microscopy Environment Remote Objects (OMERO) is an open-source image manager used by many biologists to store, organize, view, and share microscopy images, while the open-source software ImageJ/Fiji is a very popular program used to analyse them. However, there is a lack of an easy-to-use generic tool to run a workflow on a batch of images without having to download them to local computers, and to automatically organize the results in OMERO. To offer this functionality, we have built (i) a library in Java: “Simple OMERO Client”, to communicate with an OMERO database from Java software, (ii) an ImageJ/Fiji plugin to run a macro-program on a batch of images from OMERO and (iii) a new set of Macro Functions, “OMERO Macro extensions“, dedicated to interact with OMERO in macro-programming. The latter is intended for developers, with additional possibilities using tag criteria, while the “Batch OMERO plugin” is more geared towards non-IT scientists and has a very easy to use interface. Each tool is illustrated with a use case.

## Introduction

Cell biology research is a big provider of multidimensional image data through the use of multimodal microscopy approaches to decipher cellular processes. Photonic microscopes and associated areas of expertise in image analysis are often available in cellular imaging facilities which tend to propose unified tools to manage images and associated projects. Over the last decade many tools emerged which are in constant development: software platforms (QuPath, ^1^ Cell Profiler, ^2^ Icy, ^3^ KNIME, ^4^ napari ^5^), browser-based and collaborative frameworks (ImJoy, ^6^ BIAFLOWS, ^7^ TissUUmaps ^8^), and image databases (BisQUE, ^9^ Cytomine ^10^). While some of these programs are focused essentially on the analysis of images and others on their management, the global trend for image analysis in the machine and deep learning era is to use complementary software or platforms integrating a combination of these tools. In this ecosystem, the Open Microscopy Environment (OME) Remote Objects (OMERO), ^11^ and ImageJ/Fiji ^12^^–^^14^ benefit from a high level of historical implementation in the microscopy landscape involving a large community of users and developers.

OMERO is a complete platform designed for managing (organizing, editing, analysing and sharing) images online through standalone clients (OMERO.insight) and dedicated web interfaces, including a viewer for full multi-dimensional image display, a figure editor, analysis with internal scripts and an integrated data mining tool. Over 150 proprietary microscopy image formats are supported. The organisation, browsing and searching of images is eased by multiple cataloguing tools, as data can be annotated with tags, comments, key-value pairs, tables, and supplementary files.

To analyse images, OMERO also provides Application Programming Interfaces (APIs) to let developers interact with a server from programs in Java, Python, MATLAB and C++ and thus many image analysis software packages can connect to OMERO via dedicated links or plugins. ^1^^,^^2^^,^^15^^,^^16^ Among these, the official OMERO plugin for ImageJ/Fiji allows access to the OMERO.insight interface to open and treat an image on local desktops before saving regions of interest (ROIs), results tables and images in the database after a manual or semi-manual sequence of treatment, and so does the bidirectional software bridge ImageJ-OMERO in ImageJ2. Although it is easy and common to automate such processing with Fiji via a macro for a folder of images, it is not yet possible to automate all the processes (import/export, saving images, ROIs, results, etc.) through a unique Macro program in ImageJ/Fiji for images hosted in the OMERO database. The only solutions available today are either to write and execute a script in ImageJ2/Fiji similar to the example provided in the OMERO guide for threshold segmentation on datasets, or using ImageJ-OMERO.

The goal of this work is to ease the access to image analysis for all users who are managing their projects and images in OMERO. Based on a new and exhaustive library for importing/exporting images and results from and to the OMERO database, named Simple OMERO Client, which mirrors similar efforts done in Python with *ezomero* , we propose two ways to interact with the OMERO database using the ImageJ Macro language. The first one is through a graphical user interface (GUI) on the same basis as the Batch Process module of ImageJ, which will loop a macro program written for one image on whole datasets. The other one is using new OMERO Macro functions to write a macro program that will loop the analysis on datasets. Both are developed using the aforementioned Java library, Simple OMERO client, which will also be described in this paper.

## Methods

### Implementation

Simple OMERO Client a library, and two plugins, OMERO Macro Extensions and batch OMERO plugin were built. These last two, are mentioned as “plugins” as they will be new ImageJ/Fiji modules/menus to get access to (i) a vocabulary extension in macro programming to interact with OMERO, or (ii) a specific GUI to batch image analysis from OMERO. All three were written in Java 8 and use Maven ^17^ to handle their dependencies. They all rely on *ImageJ.* The two plugins depend on the *Simple OMERO Client* library which was developed to wrap calls to the underlying *OMERO Java Gateway*, its main dependency.

Simple OMERO Client

Simple OMERO Client is a Java library that we developed to factor code that was often re-used when interacting with ImageJ1 in a few projects, such as methods to retrieve pixel values or ROI data. The ImageJ-OMERO plugin, which offers similar functionality, was not used as it was aimed at ImageJ2; and not compatible with OMERO versions greater than 5.4, until recently.

Our Maven project relies on *omero-gateway*, *omero-blitz* and *omero-model* to interact with an OMERO server, but it also depends on *formats-api* to handle microscope images locally. Finally, it uses *bio-formats_plugins*, *junit4* and *Jacoco* to run tests which should ensure that the library functions as intended. These tests, however, require a local OMERO server and are ideally run through *omero-test-infra*, as is the case during the continuous integration (CI) process on the project GitHub repository, where the sources and compiled JAR can be found. The CI also uses SonarCloud and Codecov for code analysis (coverage, quality).

This library often wraps simple calls to the underlying OMERO API, but does contain complex blocks that would otherwise need to be copied to every project, such as:

• handling key/value pairs or folders,
• retrieving pixels from OMERO,
• converting ROIs between OMERO and ImageJ,
• converting ImageJ results to OMERO tables.

Most OMERO data structures are simply wrapped (Facade pattern), although a Template method pattern was also used to reinstate inheritance like in the OME model and factor common methods shared by several classes when possible (such as shapes, annotations, or hierarchy objects).

OMERO Macro Extensions

The *OMERO Macro Extensions* set of macro functions only depends on *ImageJ* and *Simple OMERO Client* to run. It relies on *junit5* and *Jacoco* for unit tests, although some functions rely on graphical elements (such as the ROI manager) and are not currently automatically tested. The CI process is similar to what is done for *Simple OMERO Client*, except code analysis which is not performed through SonarCloud or Codecov. This plugin consists of a single class implementing the *MacroExtension* interface from ImageJ: it defines 22 macro functions to interact with OMERO and acts as a front for Simple OMERO Client. To do that, it essentially parses String and Long arguments before it calls the appropriate methods from the underlying library.

Batch OMERO plugin

The *batch OMERO plugin* depends on *ImageJ* and *Simple OMERO Client* as well as *formats-api* and *bio-formats_plugins* to open images from local files. It also, optionally, relies on *scijava-common* and *scijava-ui-swing* to handle script inputs and other script languages, if possible. Currently, no automatic testing is performed and there is no CI. The main plugin window handles the connection to OMERO, displays the objects and collects the input/output while a different class is responsible for effectively running the script on all the images from the selected source and saving the results. A specific class handles the execution of the script file and collects the possible arguments beforehand: if SciJava is available, it will be used, otherwise it will fall back on ImageJ1 functions.

### Operation

To operate these tools, respective Maven dependencies need to be available, as well as a Java Virtual Machine (JVM). In practice, the OMERO dependencies can be provided by the OMERO.insight plugin for ImageJ, or better yet, by the OMERO-5.5-5.6 Fiji update site, while the bio-formats dependencies are provided by the corresponding plugin (included in Fiji).

Simple OMERO Client

The library will normally be used by developers: the easiest way is to add it as a Maven dependency to the project, as it was done by the ImageJ plugins presented in this paper. If the aim is to use an uber-JAR (including the dependencies) through another language (e.g. Python), the code just needs to be built with Maven: this will produce the desired file. This file can also be downloaded from the GitHub packages for this repository. When interacting with ImageJ, it is possible to create tables on OMERO from ImageJ results. The library also makes it possible to transfer ROIs between OMERO and ImageJ. However, as the latter only works with 2D shapes while the former handles 4D data, additional metadata are required to track which shapes belong to the same ROI. The library expects it to be done using a property in ImageJ: shapes that share the same local index for the specified key correspond to the same ROI in OMERO. Moreover, when ROIs are retrieved from OMERO, a second property, with “_ID” appended, is set with the OMERO ID as its value. Finally, tables created from ImageJ results can have a ROI column linking each line to an ROI if all the lines fulfil one of the following conditions:

• A column with the same name as the property key contains the corresponding value, and the ROI has an ID property.
• A column with the same name as the ID property contains the ROI ID.
• The label contains the name of an ImageJ ROI with those properties set.

OMERO Macro Extensions

Once the OMERO Macro Extensions plugin is installed in ImageJ, along with its dependencies, it can be used through the macro language. When writing a macro using these extensions, the first thing to do is to load the plugin with the following command: run(“OMERO Extensions”);

Connecting to OMERO is done using: Ext.connectToOMERO(“host”, 4064, “username”, “password”);

Then, switching group can be performed through: Ext.switchGroup(groupId);

Afterwards, interacting with OMERO only takes simple instructions, such as:
datasets = Ext.list("datasets");


or:
imageplusID = Ext.getImage(imageId);


When done, you can disconnect with: Ext.disconnect();

Batch OMERO plugin

**Figure 1.**
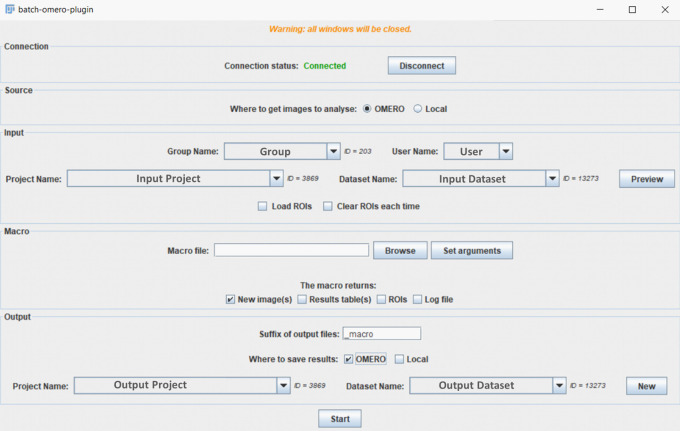
GUI window of the batch OMERO plugin.

In the same way, the batch OMERO plugin needs to be installed in the ImageJ plugins folder along with its dependencies. If this plugin is installed in ImageJ2/Fiji, it will make use of SciJava to run scripts using script parameters, otherwise it will only run ImageJ1 macro files, with arguments specified manually. When the plugin and the Simple OMERO Client are downloaded and installed, and once ImageJ/Fiji is launched, a new “Batch process…” item is added in the OMERO plugin menu. When chosen, the GUI ( Figure 1) is opened. A connection window to OMERO appears by clicking « Connect » in this window. Once connected, the drop-down menus will be filled with information coming from the default group of the User (Group, User, Project, Dataset). Depending on the saving options (OMERO or local), the window will adapt its output choices.

The tool can process images retrieved remotely from OMERO or locally from a folder, using Bio-Formats. Conversely, it can save the output on OMERO and/or locally. The main possible outputs are new images, ROIs, results tables, or log windows and the user has to specify ( Figure 1) what should be saved in accordance with the outputs of the macro.

ROIs can be loaded from OMERO: if this option is chosen ( Figure 1), then OMERO ROIs will be exported to the ROI Manager using Simple OMERO Client. 3D/4D ROIs can thus be accessed from macros through two ROI properties: “ROI” and “ROI_ID”, which contain, respectively, the local index and the ROI ID on OMERO for each 2D shape.

When saving to OMERO, the users have to choose an existing project or a dataset they own in the current group.

Furthermore, the following rules apply:

• If ROIs are saved but images are not, then they are saved to the input image on OMERO, which should be annotatable by the user.
• If images and ROIs are saved, then:
  ‐ For each image, its overlay is imported as well.
  ‐ The last active image obtained with the macro-processing gets the content of the ROI Manager, and the Results tables and log windows will be its associated files.
  ‐ If the last active image is the same than the input image but it cannot be annotated by the current user, then the image is re-imported for the user.
• If tables are saved to a project, and ROIs were loaded or saved, the tables can have a ROI column, provided they fulfil the requirements from the simple-omero-client library mentioned previously.

## Use cases

Batch OMERO plugin

**Figure 2.**
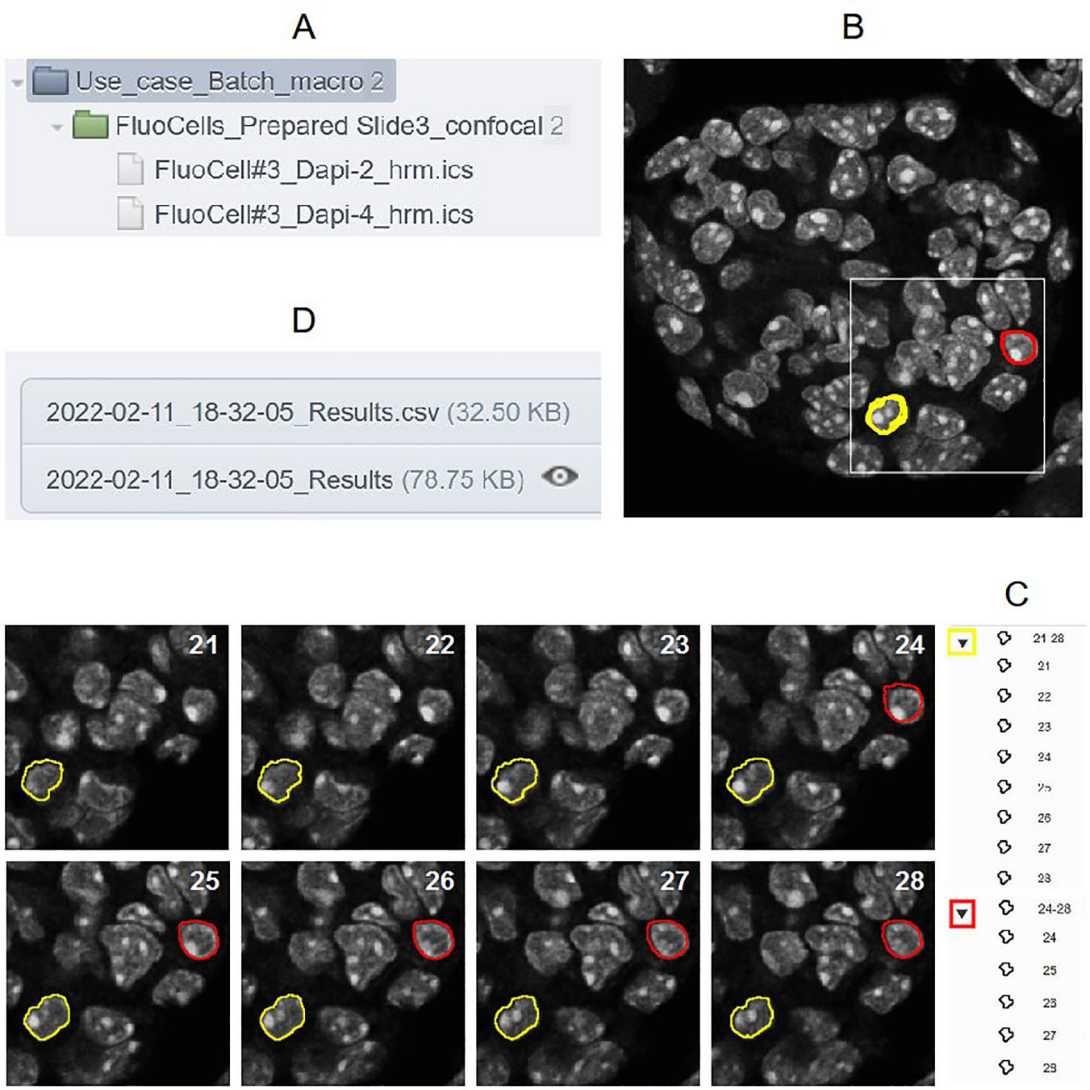
A) Project and Dataset processed by the “Macro_to_Batch_onOmero_3D” B) First image with 2 groups of ROIs displayed (red and yellow) C) View of each ROI of these groups on the different z positions of the 3D stack D) csv file and Table attached to the Project containing all the ROI measurements for all the images of the dataset processed.

As 2D or 3D segmentation is a general requirement to perform quantification in cellular biology, a use case based on this image analysis procedure is proposed to show all the possible outputs obtained with these tools. So, the batch OMERO plugin and OMERO Macro extensions were tested on a XYZ set of DAPI images obtained from a FluoCells Prepared Slide 3 (mouse kidney section with Alexa Fluor 488 WGA, Alexa Fluor 568 Phalloidin, and DAPI) from Thermo Fisher Scientific. Images were acquired on a LSM780 laser scanning confocal (Carl Zeiss, France) through a 63X/1.4 oil immersion objective (excitation 405 nm, emission 430-460 nm, voxel size 130×130×200 nm) and a blind deconvolution was applied using Huygens Remote Manager with a CMLE algorithm (Scientific Volume Imaging, Netherlands). Two macros are available to try the Batch OMERO plugin:

• “Macro_to_Batch_onOmero_3D”: this program performs 3D segmentation on the image stack of nuclei using the 3D object counter plugin ^18^ after low pass filtering and Otsu thresholding. Then ROI groups are created by gathering objects which have the same label on different slices. At the end, the macro returns the image of labels overlaid with the ROIs, a results Table ( Figure 2) and a log window. It can be run by ImageJ1 or ImageJ2/Fiji: in the former case default parameters will be used (predefined minimal sizes of objects, and all images kept at the end). In the latter, as it also uses the script parameters of ImageJ2, the minimal size of the objects can be defined by the user who can decide if images are kept or not at the end of the execution of the macro. These inputs can be displayed and modified through the GUI of the plugin with the “Set Arguments” button. This macro is the generic one called by the next one and the use case macro of OMERO Macro extensions.
• “Macro_to_Batch_onOmero_3D_IJ1_Arguments”: this macro is only coded in ImageJ1 macro language to get the parameters and calls the previous one. Its goal is to show another way to get input parameters. Indeed, the batch OMERO plugin calls the macro each time an image is opened from a dataset. If it is an ImageJ1 macro containing a dialog box to get input parameters, this one will be displayed at each execution of the macro, by default. This problem can be circumvented by getting the number of times the macro is called by the plugin with the getArguments() command. The dialog box will be displayed at the first call and parameters stored in a text file. They will be retrieved from the file at the next calls.

OMERO Macro Extensions

In this use case, the macro language extension for OMERO is used in the macro program “Test_langage_extensions_runMacro_alltags” to show another way to access and process the images through their tags. Two dialog boxes are displayed during the execution. The first one allows the user to log in and define a specific signature keyword to tag the images that will be processed (“IJ_Processed” by default). The second one contains a drop-down menu to choose the images that will be processed for 3D segmentation according to their tag, among the tags used in the default user group. If the signature tag already exists, the macro tests if it is linked to the image. If this condition is fulfilled, the image will not be processed, otherwise it will be linked to the image after processing. When the signature tag is unknown in the user group, it is created and linked to the processed image.

The plugin also includes macro templates that are conversions of some of the Groovy scripts provided in the *omero-guide-fiji* repository and illustrate the possibilities offered by the plugin.

Simple OMERO Client

The expected use case for the library is to use it as a Maven dependency in a Java project, as demonstrated by the two ImageJ plugins presented in this paper. Another use case would be to call it from an ImageJ2 script to access advanced OMERO functions directly from there. It is, for example, short and easy to retrieve the maximum value from images inside a dataset and tag them if the value is between two set thresholds (script available in OMERO toolbox examples).

## Conclusion

In this paper we provide new tools to facilitate automatic image processing with ImageJ/Fiji on images managed through an OMERO database. One of the main advantages of these tools is to simplify the macro-programming and give the opportunity to quickly analyse multiple images. Among these advantages, avoiding the tedious image format management when coding Macro programs in ImageJ/Fiji, will accelerate Macro development for end-users and help focus on the essential goal: image analysis. The spirit of the plugin could be considered close to the Batch Process plugin integrated in ImageJ1, as people without any knowledge with Macro language could record their image analysis process and apply it after on all their images. Consequently, we should highlight, that our plugin potentiates the original Batch Process by offering the choice to alternatively work with local folders, like the original one, or the files could be imported/exported from local directories or the OMERO database. The plugin and language extension approaches can be considered complementary, as the language extension will allow more complex processing, including a faster connection without GUI, a tag management of the images for example, allowing selective analyses. Another big advantage will be the delivery of CSV files and tables associated with the Project, which allows the use of the OMERO.parade data mining tool, and particularly the recent parade-crossfilter development.

## Data availability

### Underlying data

No underlying data are associated with this article.

### Extended data

The Dataset of images dedicated to these treatments and processed with the “Macro_to_Batch_onOmero_3D”, through the Batch OMERO Plugin are available, alongside their results, on the public webpage of the OMERO database from Université Côte d’Azur and EMBRC-France, managed by the “Microscopie Imagerie Côte d’Azur” (MICA) Facility and housed by “Institut Français de Bioinformatique” from: https://bioimage.france-bioinformatique.fr/omero-mica/webclient/?show=project-3006.

## Software availability

• Simple OMERO Client:
  • Software and source code available from: https://github.com/GReD-Clermont/simple-omero-client
  • Archived source code at time of publication: https://doi.org/10.5281/zenodo.6320867
  • License: GPLv2+
• OMERO Macro Extensions:
  • Software and source code available from: https://github.com/GReD-Clermont/omero_macro-extensions
  • Archived source code at time of publication: https://doi.org/10.5281/zenodo.6320876
  • License: GPLv2+
• OMERO Batch plugin:
  • Software and source code available from: https://github.com/GReD-Clermont/omero_batch-plugin
  • Archived source code at time of publication: https://doi.org/10.5281/zenodo.6367840
  • License: GPLv2+
• OMERO toolbox examples:
  • Software and source code available from: https://github.com/GReD-Clermont/omero-toolbox-examples/tree/1.0.1
  • Archived source code at time of publication: https://doi.org/10.5281/zenodo.6367851
  • License: MIT
