# Supplementary material for: Easing batch image processing from OMERO: a new toolbox for ImageJ
Source: F1000Res. 2022 Sep 12;11:392. Originally published 2022 Apr 5. [Version 2] doi: 10.12688/f1000research.110385.2 (PMC9171289; doi:10.12688/f1000research.110385.2)
Supplement: Peer review history

# Peer review: Reviewer response for version 1

**DOI:** 10.5256/f1000research.121985.r130101

**Published:** 2022-06-06

**Authors:** Caterina Strambio-De-Castillia

**Affiliations:** 1 Program in Molecular Medicine, University of Massachusetts Medical School, Worcester, MA, USA

**GENERAL NOTES**

 --------------------------

 The authors describe three software tools they have developed to facilitate the batch analysis of images that are stored and managed using the popular OMERO data management platform.

 The explicit goal described by the authors "to ease the access to image analysis for all users who are managing their projects and images in OMERO." is definitely worthwhile and the work is timely especially because similar work to connect ImageJ with OMERO ( https://github.com/imagej/imagej-omero) has not been maintained and cannot be used with recent versions of OMERO.

**SPECIFIC COMMENTS**

 --------------------------------

LANDSCAPE ANALYSIS:

 Given the potential impact and general interest of the topic and the existence of similar ongoing efforts in the community, in order to avoid confusion it is important that the authors compare their work with other tools and address the following questions:

1. Were previous efforts utilized as a starting point?
2. If not why not?
3. How are their tools better than those produced by other efforts?

CLARITY:

 We suggest that the authors might consider revising the text to increase clarity and the potential impact of their work on non-IT users.

 Specifically, the authors should consider that terms that are routinely used by software engineers such as "application", "tool", "plugin" "API" and "library" are often very confusing to biomedical researchers who are often non-computer experts.

 For example, the text in the Abstract and Introduction is a bit confusing regarding the number of tools that were developed. Below I list the primary sources of confusion in the text:

1. Line 8 of the Abstract states "we have built three tools".
2. The description in the Abstract describes three tools, called: Simple OMERO Client (a library); OMERO Macro extensions (a plugin); and Batch OMERO plugin (a plugin).
3. The last line of the Abstract states "Both tools are illustrated..."
4. The last paragraph of the Introduction describes two methods to interact with OMERO.
5. The Methods section states "Three tools were built..."

While I understand that some of the confusion arises from the fact that one of the tools is a library and the others are plugins, the authors might want to consider revising the text to ensure that biomedical researchers who are not fluent in "computerese" can understand the importance of their work.

Are the conclusions about the tool and its performance adequately supported by the findings presented in the article?

Partly

Is the rationale for developing the new software tool clearly explained?

Partly

Is the description of the software tool technically sound?

Yes

Are sufficient details of the code, methods and analysis (if applicable) provided to allow replication of the software development and its use by others?

Yes

Is sufficient information provided to allow interpretation of the expected output datasets and any results generated using the tool?

Yes

Reviewer Expertise:

Cell Biology, Microscopy, Metadata Modelling, Data Management, Imaging Science

I confirm that I have read this submission and believe that I have an appropriate level of expertise to confirm that it is of an acceptable scientific standard.

# Response

**Published:** 2022-08-08

**Authors:** Frédéric BRAU

**Affiliations:** CNRS, France

LANDSCAPE ANALYSIS:

***Given the potential impact and general interest of the topic and the existence of similar ongoing efforts in the community, in order to avoid confusion it is important that the authors compare their work with other tools and address the following questions:***

***Were previous efforts utilized as a starting point?***

 This work mixes two different axes of development: from the language part, a dedicated library developed to ease Java interaction with OMERO commands on a specific image analysis problem (NucleusJ); and from the GUI part of the batch OMERO plugin and modified groovy script from OMERO guide integrating GUI interfaces. The former library was then used to ease the Java plugins development, including the migration from groovy to Java for the batch OMERO plugin.

***If not why not?***

***How are their tools better than those produced by other efforts?***

 This tool addresses the problem from the ImageJ/Fiji point of view, to link OMERO which is more detailed in the introduction. Previous links between this database and third-party software and languages exists, as also mentioned in the manuscript. The closest approaches are dedicated groovy scripts available in OMERO guide or using the ImageJ-OMERO plugin.

CLARITY:

**We suggest that the authors might consider revising the text to increase clarity and the potential impact of their work on non-IT users. Specifically, the authors should consider that terms that are routinely used by software engineers such as "application", "tool", "plugin" "API" and "library" are often very confusing to biomedical researchers who are often non-computer experts.**

***For example, the text in the Abstract and Introduction is a bit confusing regarding the number of tools that were developed. Below I list the primary sources of confusion in the text:***

***Line 8 of the Abstract states "we have built three tools".***

***The description in the Abstract describes three tools, called: Simple OMERO Client (a library); OMERO Macro extensions (a plugin); and Batch OMERO plugin (a plugin).***

***The last line of the Abstract states "Both tools are illustrated..."***

***The last paragraph of the Introduction describes two methods to interact with OMERO.***

***The Methods section states "Three tools were built..."***

**While I understand that some of the confusion arises from the fact that one of the tools is a library and the others are plugins, the authors might want to consider revising the text to ensure that biomedical researchers who are not fluent in "computerese" can understand the importance of their work.**

 We tempted to clarify the terminology through the updated version submitted, and these points to be more accurate with the denominations between the tools mentioning that there is a library, a new set of Macro Functions through the OMERO Macro extensions plugin and a Batch OMERO plugin. These last two are mentioned as plugins as they are modules added to ImageJ/Fiji to get their functionalities, and they were developed using the library *.*

# Peer review: Reviewer response for version 1

**DOI:** 10.5256/f1000research.121985.r129917

**Published:** 2022-05-17

**Authors:** Jason R. Swedlow

**Affiliations:** 1 Division of Computational Biology, Centre for Gene Regulation and Expression, School of Life Sciences, University of Dundee, Dundee, UK

The authors introduce a set of tools to simplify access to an OMERO server from the ImageJ ecosystem (Java). Similar efforts are on-going in other programming languages e.g. https://github.com/TheJacksonLaboratory/ezomero (Python). This could maybe be mentioned to highlight the fact that the work of the authors is part of a general community effort and should be well received by scientists.

 Other groups previously released work connecting OMERO and ImageJ, see https://github.com/imagej/imagej-omero. This work has not been updated in recent years to be compatible with newer versions of the OMERO.server. Have the authors considered such work based on ImageJ2 as a starting point? If not, it will be interesting to highlight why since scientists and other software e.g. KNIME used it in some versions of their applications.

 From a technical point of view:

 The examples provided have been tested and the various GitHub repositories checked.

- Simple OMERO client. The jar associated with the tag e.g. https://github.com/GReD-Clermont/simple-omero-client/releases/tag/5.9.0 will not allow the user to run the examples. There is no indication in the README of the dependencies required. They are mentioned in the paper but it is not indicated where they can be found. The name of the omero dependencies is omero-gateway-java and not omero-gateway. Installing the uber jar simple-omero-client-with-dependencies.jar, built from source, will allow us to run the examples exposed in the README. Authors should consider uploading the uber jar to the GitHub tag and should review the installation instructions in the paper and README.
- The Authors indicate that the simple-omero-client is built and compatible with Java 8. The GitHub action only uses Java 11. Java 8 should be added to the matrix of the GitHub action to ensure compatibility.
- https://github.com/GReD-Clermont/omero_macro-extensions and https://github.com/GReD-Clermont/omero_batch-plugin, the authors should consider depending on simple-omero-client-with-dependencies.jar and not the OMERO.insight plugin. The instructions written in the paper and the ones in the README in the GitHub repository do not match. The instructions in the paper will not work since the omero-* libraries used to connect to the OMERO.server are not mentioned. The OMERO.insight plugin jar listed in the README will install several dependencies not required by the plugins or macro-extensions e.g. UI dependencies. This should be considered for multiple reasons: size required, conflict of dependencies etc. The authors should consider mentioning simple-omero-client-with-dependencies.jar or (simple-omero-client.jar +omero-* dependencies). It will also allow the authors to control the exact version of the omero libraries  e.g. the version of omero-gateway-java they want their application to depend on.
- Have the authors considered an ImageJ update site?
- Minor point:
   
  - The term Facade should be used in this context and not Façade
  - The authors should indicate that the macro extensions examples are a conversion of some of the groovy scripts, used for training scientists, in https://github.com/ome/omero-guide-fiji/tree/master/scripts/groovy. Some examples in the omero-guide-fiji repository have been requested by the scientists themselves.
  - Code example: imageplusID = Ext.getImage (imageId);. Remove space between getImage and (
  - The term fluidize in the conclusion could be replaced with facilitate

Suggestion for follow-up work. If the package https://github.com/GReD-Clermont/simple-omero-client  is going to supersede https://github.com/imagej/imagej-omero, the authors should consider implementing methods from https://github.com/imagej/imagej-omero to the new proposed package.

 Jean-Marie Burel

 Jason Swedlow

Are the conclusions about the tool and its performance adequately supported by the findings presented in the article?

Partly

Is the rationale for developing the new software tool clearly explained?

Yes

Is the description of the software tool technically sound?

Partly

Are sufficient details of the code, methods and analysis (if applicable) provided to allow replication of the software development and its use by others?

Yes

Is sufficient information provided to allow interpretation of the expected output datasets and any results generated using the tool?

Yes

Reviewer Expertise:

microscopy, cell biology, mitosis, image informatics, public data resources

I confirm that I have read this submission and believe that I have an appropriate level of expertise to confirm that it is of an acceptable scientific standard.

# Response

**Published:** 2022-08-08

**Authors:** Frédéric BRAU

**Affiliations:** CNRS, France

***The authors introduce a set of tools to simplify access to an OMERO server from the ImageJ ecosystem (Java). Similar efforts are on-going in other programming languages e.g. https://github.com/TheJacksonLaboratory/ezomero (Python). This could maybe be mentioned to highlight the fact that the work of the authors is part of a general community effort and should be well received by scientists.***

 We haven taken into account these remarks and mentioned the ezomero and ImageJ-OMERO works done before, with a similar goal, in the Introduction.

***Other groups previously released work connecting OMERO and ImageJ, see https://github.com/imagej/imagej-omero. This work has not been updated in recent years to be compatible with newer versions of the OMERO.server. Have the authors considered such work based on ImageJ2 as a starting point? If not, it will be interesting to highlight why since scientists and other software e.g. KNIME used it in some versions of their applications.***

 The library project started as a small project to wrap API calls when accessing and organizing data on OMERO from a Java program. However, as this software depended on ImageJ 1.x to open images, it soon became apparent that it would be easier to directly convert that data from OMERO to ImageJ than to create and populate basic Java objects from the server and then convert them to ImageJ. This was not the initial goal though, which is why the ImageJ-OMERO project was not considered during the project genesis. Moreover, the project focus was on making small and simple re-usable objects/methods for an IJ1 plugin: the ImageJ2 library looked impressive and using it as a starting point seemed too time-consuming considering the human resources at our disposal. In hindsight, it might have been a better choice for the community though. Merging both may still be an option, however.

***Simple OMERO client. The jar associated with the tag e.g. https://github.com/GReD-Clermont/simple-omero-client/releases/tag/5.9.0 will not allow the user to run the examples. There is no indication in the README of the dependencies required. They are mentioned in the paper but it is not indicated where they can be found. The name of the omero dependencies is omero-gateway-java and not omero-gateway. Installing the uber jar simple-omero-client-with-dependencies.jar, built from source, will allow us to run the examples exposed in the README. Authors should consider uploading the uber jar to the GitHub tag and should review the installation instructions in the paper and README.***

 Indeed, the library has only been thought as a Maven dependency, which would be available to users if they installed the other plugins following the provided steps, so instructions to install only the library to ImageJ specifically are currently missing. The uber-jar exists because when the project started, before ezomero, the library was also used to interact with OMERO from Python using JPype, as well as other Java programs outside of ImageJ. However, using it in ImageJ may cause conflicts if the official OMERO.insight plugin is installed, unless dependencies are “shaded” and “relocated”, which is why it was not readily available. The aim will be to rely on the ImageJ-OMERO-5.5 update site in Fiji to provide the dependencies and, alternatively, the uber-jar for ImageJ1. Instructions will be updated when this solution will be operational.

***The Authors indicate that the simple-omero-client is built and compatible with Java 8. The GitHub action only uses Java 11. Java 8 should be added to the matrix of the GitHub action to ensure compatibility.***

 The paper states the language level in which the library is written is 8, it is true though that the inference would be that it is therefore built and compatible with Java 8, when it only uses Maven properties to convey this information to the Java 11 compiler (source = 8, target = 8), which could fail in theory. However, as the build is done through omero-test-infra, it is the Dockerfile that defines which JDK is used for the build. It will be changed to 8 in 5.9.2. The GitHub action JDK will remain 11 though, as it is required for SonarCloud and not used for compilation.

https://github.com/GReD-Clermont/omero_macro-extensions and https://github.com/GReD-Clermont/omero_batch-plugin, the authors should consider depending on simple-omero-client-with-dependencies.jar and not the OMERO.insight plugin. The instructions written in the paper and the ones in the README in the GitHub repository do not match. The instructions in the paper will not work since the omero-* libraries used to connect to the OMERO.server are not mentioned. The OMERO.insight plugin jar listed in the README will install several dependencies not required by the plugins or macro-extensions e.g. UI dependencies. This should be considered for multiple reasons: size required, conflict of dependencies etc. The authors should consider mentioning simple-omero-client-with-dependencies.jar or (simple-omero-client.jar +omero-* dependencies). It will also allow the authors to control the exact version of the omero libraries e.g. the version of omero-gateway-java they want their application to depend on.

 Instructions in the paper should indeed reflect what is in the README. Moreover, what is in the README may need adjustments, depending on the possible use of the library uber-jar. As mentioned previously, the procedure in the README (OMERO.insight plugin + library jar + plugin jars) was chosen to prevent conflicts between OMERO.insight and the library when it comes to dependencies.

***Have the authors considered an ImageJ update site?***

 An update site has been considered but OMERO dependencies need to be properly handled beforehand. If the plugins can rely on the ImageJ-OMERO update site (and offer to activate it), then an update site for these JARs will be set up.

 The Minor points have been considered and the corresponding corrections were applied in the new version of the manuscript.
